# *In-situ* visualization of sound-induced otolith motion using hard X-ray phase contrast imaging

**DOI:** 10.1038/s41598-018-21367-0

**Published:** 2018-02-15

**Authors:** Tanja Schulz-Mirbach, Margie Olbinado, Alexander Rack, Alberto Mittone, Alberto Bravin, Roland R. Melzer, Friedrich Ladich, Martin Heß

**Affiliations:** 1 Ludwig-Maximilians-University Munich, Department Biology II, Zoology, Großhaderner Straße 2, 82152 Planegg-Martinsried, Germany; 2 European Synchrotron Radiation Facility (ESRF, ID19), 71 Avenue des Martyrs, 38000 Grenoble, France; 3 European Synchrotron Radiation Facility (ESRF, ID17), 71 Avenue des Martyrs, 38000 Grenoble, France; 4 Bavarian State Collection of Zoology (ZSM), Münchhausenstraße 21, 81247 Munich, Germany; 5 University of Vienna, Department of Behavioural Biology, Althanstraße 14, 1090 Vienna, Austria

## Abstract

Regarding the basics of ear structure-function relationships in fish, the actual motion of the solid otolith relative to the underlying sensory epithelium has rarely been investigated. Otolith motion has been characterized based on a few experimental studies and on approaches using mathematical modeling, which have yielded partially conflicting results. Those studies either predicted a simple back-and-forth motion of the otolith or a shape-dependent, more complex motion. Our study was designed to develop and test a new set-up to generate experimental data on fish otolith motion *in-situ*. Investigating the basic parameters of otolith motion requires an approach with high spatial and temporal resolution. We therefore used hard X-ray phase contrast imaging (XPCI). We compared two anatomically well-studied cichlid species, *Steatocranus tinanti* and *Etroplus maculatus*, which, among other features, differ in the 3D shape of their otoliths. In a water-filled tank, we presented a pure tone of 200 Hz to 1) isolated otoliths embedded in agarose serving as a simple model or 2) to a fish (otoliths *in-situ*). Our new set-up successfully visualized the motion of otoliths *in-situ* and therefore paves the way for future studies evaluating the principles of otolith motion.

## Introduction

In teleost fish, otoliths are solid calcium carbonate biominerals overlying the respective sensory epithelium in the inner ear^1^. Otoliths, especially those of the saccule, show a species-specific shape^2^ and are about three times denser (approx. 2.93 g cm^−3 ^^3^) than the soft tissue including the sensory epithelium (e.g. muscle tissue, approx. 1.05 g cm^−3 ^^4^). If the fish moves in a sound field, the denser otolith lags behind relative to the movement of the sensory epithelium^1^. The shearing forces deflect the ciliary bundles of the sensory hair cells, which leads to maximum stimulation of the hair cell if the bundle is pivoted into the direction of the kinocilium^5^. Hence, otoliths help fish to detect gravitational forces, gauge linear acceleration triggered by active motion (swimming) or register passive motion induced in a sound field^6^. A deeper knowledge about the functional role of otoliths in fish ears is essential for a better understanding of hearing. This is also an important step in attempting to predict the effects of ocean acidification or dense crowding in aquacultures on otolith biomineralization and thus on potential alterations in fish hearing^7^.

Otoliths vary greatly in mass and shape among teleost species^2,8^. This raises the question of how these characters affect the relative motion between otolith and sensory epithelium and the stimulation of sensory hair cells^9,10^. We currently know little about the motion itself (e.g. direction, amplitude) or about factors influencing this motion^10,11^. Theoretical considerations^12–16^ supported by experimental evidence^17^ indicate that the fish ear is sensitive to particle motion. The otolith–sensory epithelium system acts as an accelerometer because particle acceleration is the relevant stimulus for the otolith organs in the fish ear^6,17^.

First experimental studies^18^ indicated a simple forward and backward motion between the otolith and sensory epithelium. This was adopted for initial mathematical modelling^19,20^. Those studies modelled otoliths as spheres, whereas theoretical considerations strongly assumed a shape-dependency of the movement^11,21^. A laser vibrometer study on exposed perch saccules^22^ provided first experimental evidence that otolith shape might influence otolith motion. Saccular otoliths in perch “vibrated” as the central and marginal portions of the otoliths displayed different motion patterns in terms of different relative velocities. At a stimulus frequency of 220 Hz (horizontal sinusoidal vibration, 630 µm/s) the vertical movement at the otolith center amounted to ca. 50–60 µm/s which was within the range of the “background” movement of the skull (30–60 µm/s) whereas motion at the anterior and posterior margins was ca. 120–140 µm/s^22^. Recent mathematical modelling using finite element analysis supported the assumption of shape-dependent otolith motion; a more complex model shape (hemisphere) distinctly deviated from a simple back-and-forth motion compared to the motion of a modelled spherical shape in a sound field assuming a 200 Hz stimulus (ref. ^23^see also^24–26^).

Data from experimental studies are rare, and those investigations were conducted under artificial conditions such as applying strong centrifugal forces on fish heads (up to 11 g^18^) or opening the skull and exposing the ears^22^. Synchrotron radiation imaging techniques are promising to provide new experimental data that are needed to test existing hypotheses on otolith motion and to provide new input data for advanced mathematical modelling. Ideally, otolith motion should be observed and quantified in a living animal during sound presentation. Recent studies on the biting and chewing mouthparts of living cockroach *Periplaneta americana*^27^ or flying blowfly^28^ using synchrotron radiation imaging techniques stimulated our idea that movement of otoliths provoked by a sound stimulus could be visualized *in-situ* as well.

In the following, we outline how we designed and developed the set-up for successfully investigating otolith motion at the European Synchrotron Radiation Facility (ESRF, Grenoble, France) at the beamlines ID17 (biomedical beamline; ID = insertion device) and ID19 (microtomography beamline). We further discuss the methodological potential of this technique along with current limitations and issues that need to be addressed in future studies.

## Material and Methods

### Animals

We chose two cichlid species, the orange chromide *Etroplus maculatus* (wild type) and the slender lion head cichlid *Steatocranus tinanti* (wild type). These two species differ in the size of the swim bladder, the relationship between swim bladder and inner ears, and the overall shape of the saccular otolith. In *E*. *maculatus*, the swim bladder is large contacting the inner ears through a bipartite structure of the anterior swim bladder extensions^29^, whereas *S*. *tinanti* exhibits a vestigial swim bladder that has no connection to the ears^30^. The saccular otoliths in *E*. *maculatus* are pentagonal and distinctly curved, whereas those in *S*. *tinanti* are fusiform and less curved^31^.

Both species have been well investigated with regard to the morphology of the inner ears and the swim bladder as well as their auditory abilities in terms of relative auditory thresholds and the detectable frequency range^29–31^. This profound morphological knowledge was important because the 2D radiographic imaging of whole fishes shows all skull bones and otoliths of the left and right body sides overlying each other, making it difficult to distinguish between different structures without such prior knowledge.

In total, six fish (*E*. *maculatus*) ranging in standard length from 53 mm to 63 mm, and four saccular otoliths from *E*. *maculatus* and *S*. *tinanti* embedded in agarose (two samples in 0.5% and two in 1% agarose, preparation see below) were investigated. Only outcomes of those samples – i.e. one whole specimen of *E*. *maculatus* and two otoliths embedded in 1% agarose (one from *E*. *maculatus* and one from *S*. *tinanti*) – are presented which were analyzed in the final set-up at ID19 with a pixel size of 6.5 µm (Table 1). The other samples served in optimizing the set-up itself (at ID17 and ID19).

**Table 1:** Overview of samples and measurements.

Species	Otolith in agarose	Otoliths *in-situ*	SL (mm)	Otolith mass (g)	Otolith volume (cm^3^)	Otolith density (g/cm^3^)	2D	tomography
*Etroplus maculatus*		x	53		UO left: 0.000163 right: 0.000151 SO left: 0.00104 right: 0.00099 LO left: 0.0000551 right: 0.0000540		x	x (ZSM)
		right UO	53					x (ID19)
	right SO		52	0.002294	0.000772	2.97	x	x (ZSM)
*Steatocranus tinanti*	left SO		83	0.001224	0.000396	3.09	x	x (ZSM)

ID19, ESRF beamline ID19; LO, lagenar otolith; SO, saccular otolith; SL, Fish standard length; UO, utricular otolith; ZSM, Bavarian State Collection of Zoology Munich.

Fish (*E*. *maculatus*, wild type) subjected to the experiments testing otoliths *in-situ* were purchased from French aquarium traders and kept at the animal care facility of the ESRF. The animals (Table 1) were maintained in two 3 to 5l-plastic tanks under a 12:12 h light-dark cycle and fed once a day with commercial flake food. In each tank, a bubble stone was used to aerate the water. The two otoliths of *E*. *maculatus* and the two otoliths of *S*. *tinanti* embedded in agarose were re-analyzed from a former study^31^.

All experiments at the ESRF were conducted in accordance to ethical guidelines. As no living animals were tested during the imaging procedure, no ethical approval was required.

### Set-up design

The set-up design, especially with regard to the test tank dimensions, was developed at ID17. The stage at ID17 can handle weights up to 10 kg, which was important because we initially aimed to use a tank volume as large as possible. At ID19, we refined the set-up and performed the final 2D radiographic imaging. Here, the frame rate could be adjusted to the sound stimulus of 200 Hz due to access to a higher photon flux density. This yielded shorter exposure times and thus an increased temporal resolution at that beamline.

Initially, we tested a large cylindrical Plexiglas® tank (inner diameter: 22 cm; wall thickness: 5 mm; volume: 9.5 l) with an underwater speaker (UW-30, Ela Data GmbH, Germany; max. diameter 18.3 cm) suspended from above at ID17. The large water body, however, caused considerable attenuation of the beam (0.87% transmission in the center of the tank). Moreover, the curved wall of the cylindrical tank also negatively affected the achievable spatial and temporal resolutions and the signal-to-noise-ratio. Even though the larger tank would have been preferable due to its better acoustic properties, we conducted the further experiments with a smaller rectangular Plexiglas® tank (inner dimensions: 10 cm × 10 cm × 20 cm; wall thickness: 5 mm; volume: 2 l) and a small underwater speaker (Daravoc MA001, max. diameter 6.7 cm) suspended from above without touching the tank walls. Still, the remaining water body and the tank walls absorbed considerable amounts of the beam (12.75% transmission). Hence, we used hard X-ray phase contrast imaging (XPCI), focusing on 2D radiography instead of performing tomography. Test subjects were tested in two orientations; sound impinged 1) on the dorsal part of the mounted otoliths or fish head (xyt) or 2) on the medial otolith face or right body side of the fish (xzt). Here, x describes the motion along the rostro-caudal, y the motion along the dorso-ventral, and z the motion along the medio-lateral axes depending on time t.

At ID17, we used a pixel size of 6.2 µm, a frame rate of 124.98 fps (temporal sampling; at ID17, this was the maximally feasible frame rate for our set-up to achieve acceptable signal-to-noise ratios, SNRs) with an integration time of 1 ms and a photon energy of 60 keV (monochromatic beam, DeltaE/E approx. 10^−4^). The detector was a PCO.Edge.5.5 connected to a 1x optical magnification system guiding to the detector the light produced by the LuAG fluorescent screen (for details see^32^).

At ID19, we used similar settings (pixel size: 6.5 µm; integration time: 1 ms; photon energy: 64 keV). In order to gain sufficiently high photon flux density, the beamline was operated in a pink configuration: the white radiation from a wiggler insertion device was only filtered by Al, C and Cu attenuators as well as the mandatory Be window in the optical beam path. As consequence, a narrow-bandwidth pink illumination was accessible which was characterized by a high amount of photons and a homogeneous wave front for sensitive phase-contrast imaging. As detector for fast micro-radioscopy an indirect system was used: two photo-lenses in tandem-design (face-to-face, with an effective 2.1x magnification, Hasselblad) were used to project the luminescence image of a LuAG:Ce (Ce-doped Lu_2Al_5O_12) single-crystal scintillator onto the CMOS sensor of a commercial camera (type: pco.dimax, PCO AG, Germany).

With the finally adjusted frame rate of 198.02 fps at ID19, i.e. 198.02 frames per second acquisition, the frames were acquired every 5.05 ms, while the period of the 200 Hz sound stimulus corresponded to 5.00 ms (1/200 s^−1^). With this slight mismatch of 0.05 ms between image acquisition rate (frame rate) and the period of the sound stimulus, we should sample approximately every tenth oscillation of otolith motion, given that the otoliths and other structures (e.g. bones, swim bladder) moved according to the sound stimulus with a frequency of 200 Hz. We thus predicted to observe a virtual otolith motion of 2 Hz.

**Figure 1:**
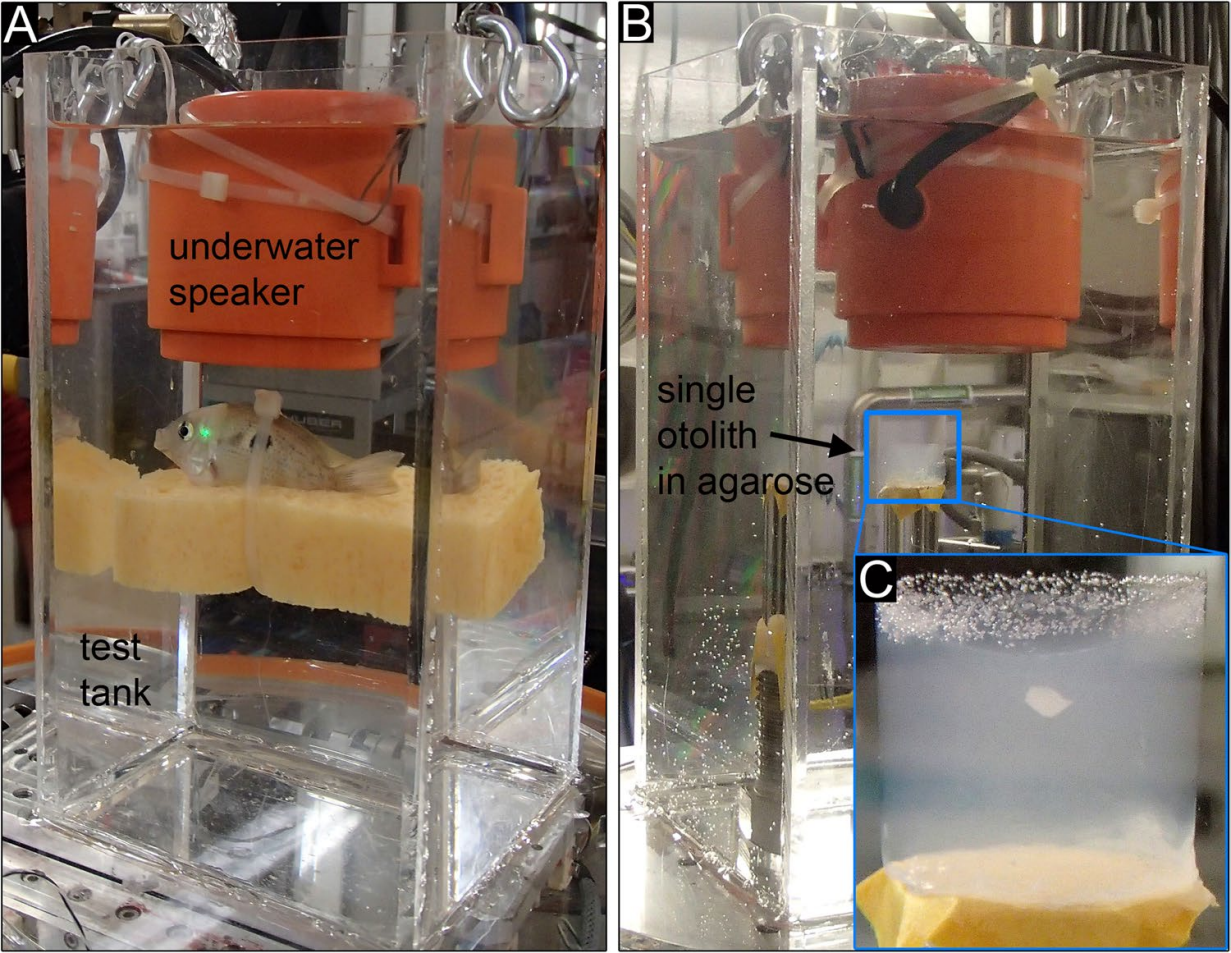
Set-up for the 2D radiographic experiments using (**A**) a fresh-dead fish or (**B**) an isolated otolith embedded in 1% agarose as test subjects. The inset in (**C**) shows the isolated right saccular otolith of *E*. *maculatus* with sound impinging on the dorsal otolith margin.

Besides the whole fish, we additionally studied isolated otoliths embedded in small cylinders (ca. 1.5 ml) of 0.5 or 1% agarose (see Fig. 1). The otolith-in-agarose samples provided a simple model to test the efficiency and success of visualizing otolith motion using different set-up conditions, especially when adjusting the frame rate.

### Acoustics

#### Sound presentation and characterization of tank acoustics

**Figure 2:**
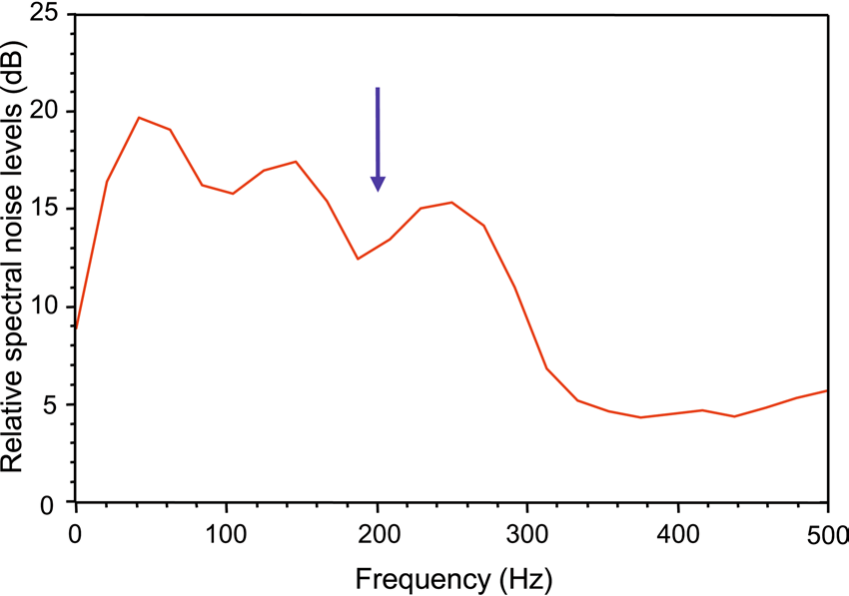
Spectrum of ambient noise measured in the rectangular test tank at ID17. Note that the sound pressure level of the 200 Hz stimulus (158 dB re 1 µPa) was distinctly above that measured for the background noise (107 dB re 1 µPa). The blue arrow indicates the stimulus frequency.

In our experiments, we focused on a 200 Hz pure tone stimulus for two reasons. Even though the two cichlid species exhibit a different morphology of the saccular otolith, they show similar auditory sensitivities in the frequency range of 100 to 300 Hz^30^. Moreover, we wanted to compare our data with outcomes obtained in studies modelling otolith motion to a 200 Hz stimulus^23^ and an experimental study using, among others, a 220 Hz stimulus^22^. To ensure that the potentially observed movement of the otoliths is not only provoked by background noise in the hutch, we modified the stimulus as follows. The final stimulus (total duration: 9 s) consisted of a no-sound period (2 s) followed by the actual 200 Hz pure tone (sound period, 5 s) again followed by a no-sound period of 2 s. This stimulus was presented in three consecutive repeats. The sound stimulus was generated using the software CoolEdit 2000 (Syntrillium Software Corp. Phoenix, AZ) and the internal sound card of a laptop connected to the Daravoc underwater speaker via an amplifier (36B2, constructed at the University of Vienna). Sound pressure levels (SPLs, LLSP, L frequency weighting, S time weighting) of the generated sound were measured using a miniature hydrophone (Brüel & Kjær 8103, sensitivity: −211 dB re 1 V/µPa) connected to a sound level meter (Brüel & Kjær 2238 Mediator) which was calibrated using a hydrophone calibrator (Brüel & Kjær 4229). The hydrophone and sound level meter were also used to measure the SPLs of ambient noise in the test tank at both beamlines (LLeq, L frequency weighting, recording duration: 1 min). The ambient noise was recorded with the miniature hydrophone connected to the laptop using the software CoolEdit 2000. The ambient noise spectrum (see Fig. 2) was then analyzed with the STX sound analyzing software (Acoustics Research Institute, Austrian Academy of Sciences, Vienna, Austria).

#### Evaluation of particle acceleration levels

The sound field in the rectangular Plexiglas® tank was characterized by measuring the SPL directly and calculating the particle acceleration level (PAL) in a walk-in soundproof room at the Department of Behavioural Biology, University of Vienna. The SPL (LLSP) was measured with the miniature hydrophone connected to the sound level meter. The SPL generated by the underwater speaker (Daravoc MA001) when playing back 200 Hz pure tones during these tests equaled the SPL measured during experiments at the beamline ID19. In order to determine PAL in the vertical direction, a calibrated underwater miniature acoustic pressure-acceleration (p-a) sensor (S/N 2007–001, Applied Physical Sciences Corp., Groton, CT) was placed 3 cm below the underwater speaker. The p-a sensor consists of two built-in units: a piezoelectric, omnidirectional hydrophone (sensitivity: −173.7 dB re 1 V/µPa) and a bi-directional accelerometer (sensitivity: −137.6 dB re 1 V/µm/s²). The p-output of the sensor was connected to the sound level meter and yielded a SPL close to that of the B&K hydrophone (difference 2 dB) when corrected for the difference in sensitivity between both pressure sensors. Subsequently, the a-output of the sensor was connected to the sound level meter and the level was corrected according to the difference in sensitivity between the p- and a-units of the p-a sensor. SPLs were calculated in dB re 1 µPa and PALs in dB re 1 µm/s². These are the international units for sound pressure and particle acceleration according to ISO standards (ISO 1683, 1983^33,34^).

### Sample preparation

#### Otoliths *in-situ*

Prior to the experiment, the fish was anaesthetized and euthanized with an overdose of neutral-buffered 0.4% tricaine methanesulfonate (MS-222, Sigma Aldrich, France). Subsequently, the standard length was measured to the nearest millimeter using a caliper. After the opercula movements had clearly ceased (approx. after 20 min) due to treatment with MS-222, the fish was fixed in the test tank with a piece of foam rubber and a cable tie with (1) the dorsal side up (Fig. 1A) and subsequently, with (2) the right body side pointing toward the underwater speaker. After the experiment, the fish was fixed in 10% neutral-buffered formalin for further studies at the MicroCT Nanotom at the Bavarian State Collection of Zoology (ZSM, Munich, Germany).

#### Isolated otoliths embedded in agarose

Weight measurements of the isolated otoliths used in the “agarose experiments” were taken from a previous study in which these otoliths had been investigated^31^. Then, isolated saccular otoliths were embedded in either 0.5% or 1% agarose (Agarose Standard for electrophoresis, Carl Roth, Germany) using a multi-well plate (maximum volume per well: 1.5 ml; Fig. 1B). Imaging of isolated otoliths embedded in 1% agarose provided a more robust set-up than those embedded in 0.5% agarose. In the 0.5% agarose, numerous air bubbles quickly emerged during the scanning procedure and impeded imaging of otolith motion (especially at ID19). All subsequent experiments were therefore performed with otoliths embedded in 1% agarose, which showed less bubble formation. Bubbles were used to test indirectly whether the embedded otolith simply moved together with the agarose block or if differential movement between otolith and surrounding medium occurred. Hence, we also evaluated the motion of bubbles that formed in the second set of experiments, i.e. when sound impinged on the medial (sulcal) otolith face.

### Imaging

#### 2D radiography: Image processing

Images were processed using ImageJ v. 1.51n^35^. The 16-bit (unsigned) mode of the original images was retained and the background was removed by adding the inverted reference image from each image in the stack. The respective reference image had been recorded at the same pixel size, frame rate, and integration time as the image stack without, however, test subject or sound presentation. In one case (isolated otolith of *E*. *maculatus* in “lateral view”), the canvas size of the inverted reference image had to be increased by adding four pixels to the top of the image to compensate for a vertical shift between reference image and the images in the stack. After background correction, brightness and contrast were enhanced and all images of the stack were flipped vertically and/or horizontally to account for virtual flips of the test subject during imaging. Subsequently, images were cropped from an original image size of 1008 × 1012 pixels to a size of e.g. 550 × 360 pixels to avoid displaying “empty” background.

#### Image Analysis – Characterizing otolith motion and displacement

**Figure 3:**
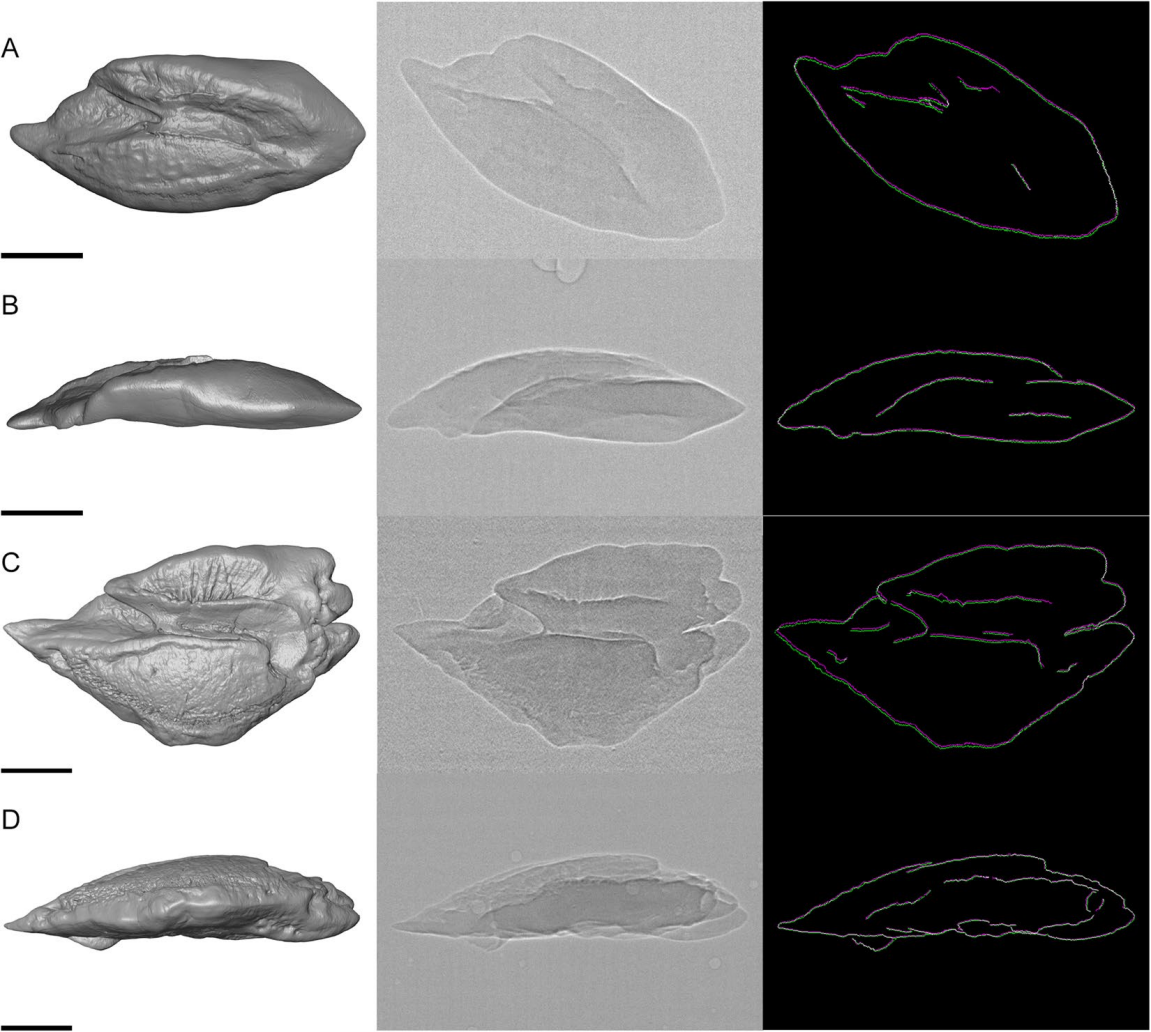
Saccular otoliths of *S*. *tinanti* (**A**,**B**) and *E*. *maculatus* (**C**,**D**) used for the experiments in 1% agarose shown as right otoliths. (**A**,**B**) represent mirror images of the left otolith of *S*. *tinanti*. The first column shows 3D reconstructions of the otoliths in medial (**A**,**C**), dorsal (**B**), or ventral (**D**) views. 2D radiographic images of the same otoliths are shown in the middle column. The right column displays the overlays of averaged maximum (purple) and minimum (green) positions of the otolith during otolith motion due to stimulus presentation. Scale bars, 500 µm.

**Figure 4:**
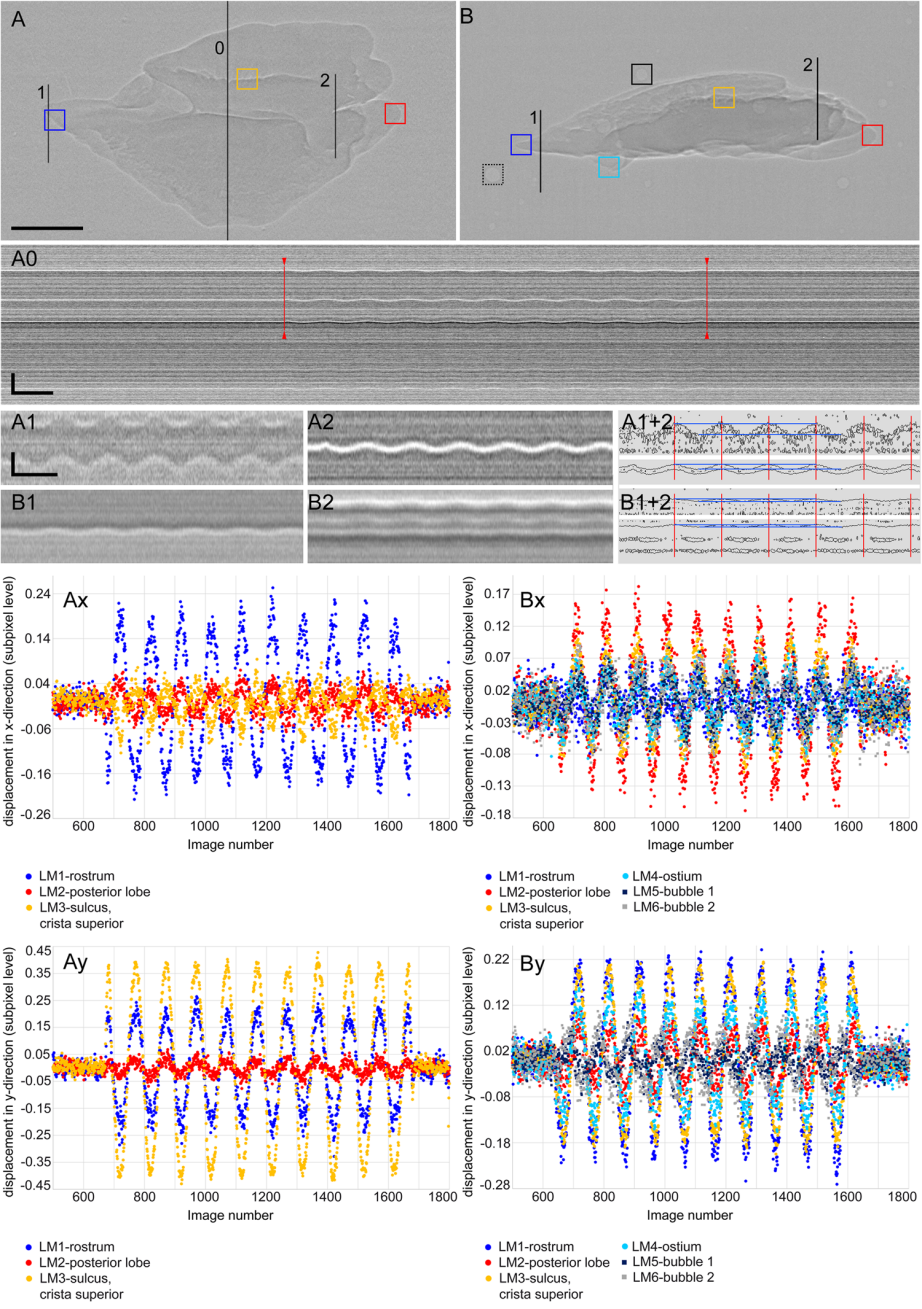
Motion of the saccular otolith of *E*. *maculatus* illustrated by yt-reslicing (**A0**–**A2**, **B1**–**B2**) and residual plots of “displacement” versus image number (**Ax**-**y**, **Bx**-**y**) obtained by the template matching procedure in ImageJ v 1.51n. “A” always indicates sound impinging on the otolith’s dorsal margin, “B” on the otolith’s medial face. Vertical lines in (**A**) and (**B**) indicate the position of yt-reslicing shown in (**A0**, **A1**–**A2**), and (**B1**–**B2**). In (**A0**), the otolith motion is detectable only during the stimulus presentation (regular oscillating pattern; begin and end of the oscillation indicated by red arrow heads and lines), whereas in the silent periods before and after the stimulus presentation no movement is observed (“flat lines”). (**A1**–**A2**) and (**B1**–**B2**) reveal that the anteriormost tip (rostrum) of the otolith shows greater displacement than the posterior lobe (**A1** vs. **A2**, **B1** vs. **B2**) and that displacement is larger when sound impinged on the dorsal margin vs. the medial otolith face (**A1–A2** vs. **B1**–**B2**). According to the residual plots, all regions of the otolith move in phase in both views (**Ay**, **Bx-y**; exception: motion along the x-axis with sound impinging on the otolith’s dorsal margin, **Ax**). The two bubbles and the otolith move out-of-phase along the y-axis (**By**). Scale bars in (**A0**–**A1**), vertical bars, 250 µm (**A0**) and 50 µm (**A1**), horizontal bars, 500 ms. Vertical distance between the pairs of blue lines from top to bottom in (**A1**–**A2**), 32.5 µm and 15.6 µm, in (**B1**–**B2**), 7.8 µm and 6.5 µm.

To illustrate otolith motion, we produced overlays of the mean outlines of the respective otolith at the nine minimum and nine maximum positions during the motion period as averaged otolith outlines (Fig. 3: right column). We also applied the “Reslice YT” tool in ImageJ v. 1.51n at transects in anterior, central, and posterior positions of the otoliths (Fig. 4A0–2,B1–2) as well as the ImageJ plugin “Template Matching – Align slices in stack”.

The template matching procedure corrects shifts between images within a stack using a selected region of interest (ROI) as template for slice alignment. The corrections performed in x- and y- directions for each image can be saved as x-y-coordinates. We ran this procedure with the normalized correlation coefficient method in which a match between the template (selected ROI) and a corresponding structure on the subsequent images depends on the relative intensity contrast of the pixels under the template^35^. Further, we chose a uniform size for all selected ROIs (28 × 28 pixels), the subpixel registration activated, and a search area around the ROI set to 10 pixels. Note that this displacement does not necessarily reflect the true amplitude in micrometers of the moving structure within the ROI because structures within ROIs highly contrasted against the background may show a higher “amplitude” than those that display similar gray values compared to the background.

At most studied ROIs, the raw plots illustrating the “displacement” in x- or y-directions versus image number showed a linear trend. To facilitate comparison between different ROIs and to identify phase shifts, linear trends were removed for all ROIs in the software PAST v. 3^36^ using the “Transform – remove trend” method and then plotting the residuals. We studied between three and 12 ROIs per sample. In all samples and for both orientations, we analyzed the ROIs of the first and the third recording to test whether the motion of otoliths or other structures (bones, swim bladder) showed a reproducible pattern.

The maximum displacement of otoliths, bones and anterior swim bladder extensions in x- and y-directions in micrometers was evaluated from the yt-reslices, and by using the outcomes of the template matching procedure.

The reslices were increased by 5 times in y-direction and the distance between minimum and maximum displacement was evaluated at subpixel resolution by creating “contour plots” (see Fig. 4A1–2,B1–2) with a line thickness of 1 pixel in Adobe Photoshop® CS6. For conversion into true amplitudes in micrometers, the values were divided by the factor 5 to correct for the artificial increase and then multiplied by the pixel size of 6.5 µm.

Using template matching, the image numbers of minima and maxima at the ROIs were inferred from the residual plots. Then, the x-y-values of a distinctive landmark such as the anterior- or posterior-most tips of the saccular otolith within or close to the ROI were determined in the images of minimum and subsequent maximum displacements. The respective difference of x- or y-values was calculated in pixels and then conversed into micrometers because pixel size was 6.5 µm.

#### Tomography

**Figure 5:**
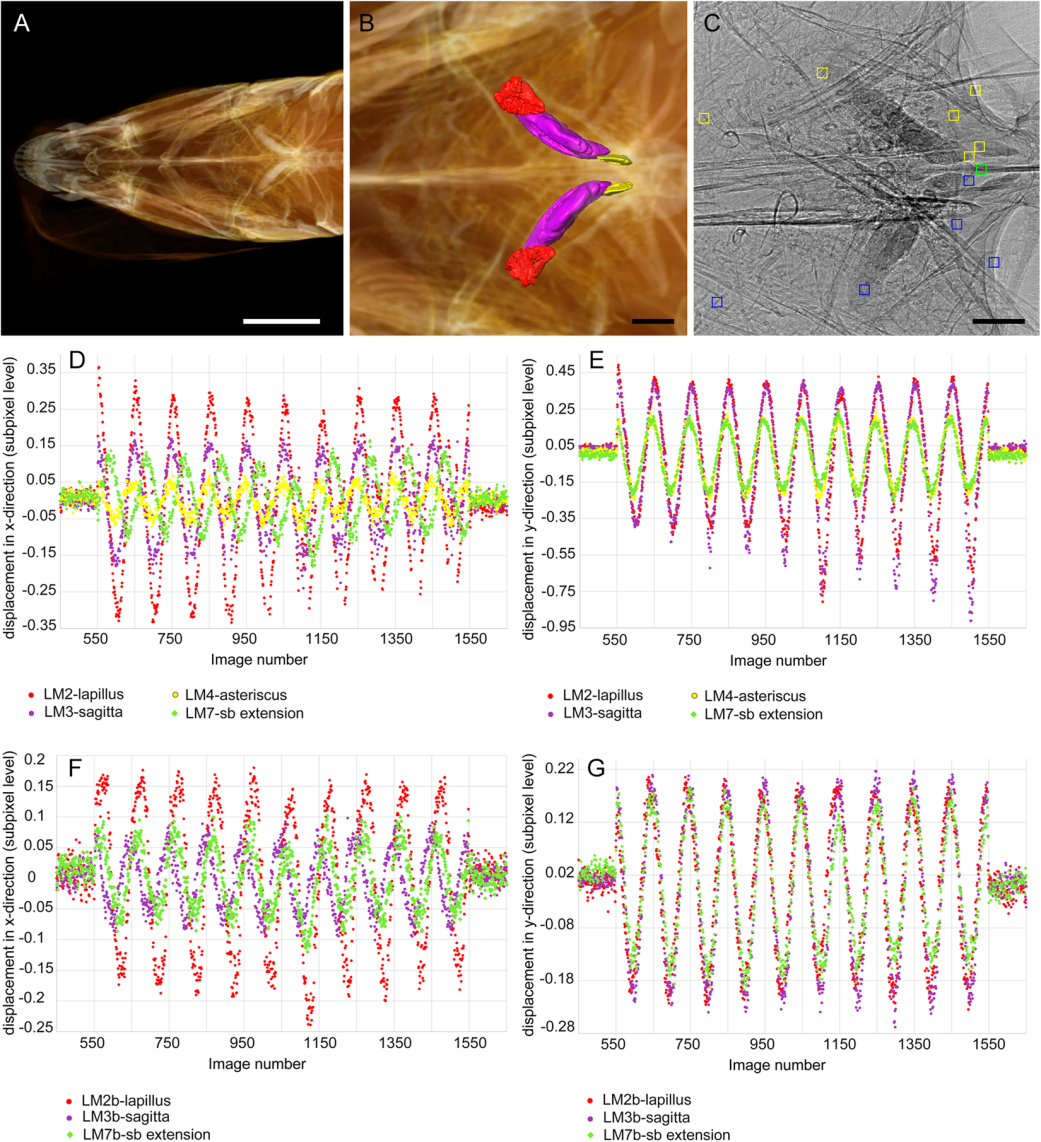
Motion of otoliths *in-situ* in *E*. *maculatus* with sound impinging on the right body side of the fish. 3D reconstructions in (**A**,**B**) illustrate the position of the utricular (red), saccular (purple) and lagenar (yellow) otoliths *in-situ*. (**C**) 2D radiographic image indicating the selected regions for template matching (yellow boxes: right body side, blue boxes: left body side, green box: central structure, i.e. the parasphenoid). Motion is illustrated with residual plots of “displacement” versus image number (**D**–**G**) obtained by the template matching procedure in ImageJ v 1.51n. Plots in (**D**,**E**) show moving structures of the right body side, those in (**F**,**G**) moving structures of the left body side. For both body sides, otoliths and anterior swim bladder extension move out-of-phase along the x-axis (**D**,**F**). Along the y-axis, all structures move in phase on the left body side (**G**), but slightly out-of phase on the right body side (lagenar otolith and swim bladder vs. utricular and saccular otolith; **E**). Scale bar in (**A**), 1 cm, scale bars in (**B**,**C**), 500 µm.

Samples were scanned with the microCT Nanotom at the Bavarian State Collection of Zoology (ZSM, Munich, Germany) to generate high-resolution 3D reconstructions of the isolated otoliths used in the agarose experiments, and to provide 3D models of the whole fish in order to identify bones that overlie the otoliths seen in the 2D radiographic experiments (Fig. 5A–C).

The right saccular otolith of *E*. *maculatus* and the left saccular otolith of *S*. *tinanti* used in the agarose experiments were scanned with an isotropic voxel size of 1.830 µm using a focal spot size of 1.8 µm, 90 kV, 170 µA, and an integration time of 750 ms generating 1440 raw 16-bit images. The whole fish of *E*. *maculatus* was scanned using a voxel size of 11.465 µm with 80 kV, 120 µA, and an integration time of 500 ms.

The utricular otolith (lapillus) in *E*. *maculatus* is characterized by a distinct radial arrangement of rod-shaped crystal regions in the anterior portion^31^, which was less contrasted in the 2D radiographs. We therefore performed a high resolution *in-situ* phase contrast tomography of a right utricular otolith of another fresh-dead individual at ID19. The utricle and its otolith were scanned with a voxel size of 0.65 µm, at 19 keV, scan duration 2.8 min, and a scan range of 180°.

Cropped 8-bit image stacks (scans at the ZSM) or the 16-bit image stack (scan at ID19) were further processed in Amira v. 6.2 and 6.3. Otoliths were labelled in each slice using a threshold-based segmentation for surface rendering (Fig. 3: left column); manual labelling and correction were performed, if necessary, in combination with volume renderings (Fig. 5A,B). Subsequently, otolith volumes were determined from the final otolith surface models in Amira v. 6.3.

### Data availability statement

Datasets generated and analyzed during the current study are available from TSM on reasonable request.

## Results

### Tank acoustics

In the rectangular test tank, the ambient noise (background) spectrum revealed highest relative amplitudes in the low frequency range at about 300 and 550 Hz (Fig. 2). The SPLs of ambient noise of 107 dB (ID17) or 119 dB (ID19) were distinctly lower than the SPLs of 158 dB (ID17) or 157 (ID19) measured during the presentation of the sound stimulus. The evaluated PAL was 130 dB re 1 µm/s^2^ in vertical direction and 124–125 dB re 1 µm/s^2^ in horizontal directions resulting in 4.0 µm of water displacement in vertical and 2.0–2.3 µm in horizontal directions. The calculated water displacement during sound stimulation is 0.04 µm when applying the equation “displacement = pressure amplitude/(density of water * sound speed of water * frequency * 2 * pi) = 70.8 Pa [ = SPL of 157 dB re 1 µPa]/(1,000 kgm^−3^ * 1,449 ms^−1^ * 200 s^−1^ * 2 * pi)” (cf.^15^).

### Sound-induced otolith motion

The recordings of the no-sound periods before and after the actual sound stimulus presentation revealed that otoliths (Fig. 3) moved exclusively during the period of sound presentation (Fig. 4A0). Otoliths in agarose and *in-situ*, as well as bones and the anterior swim bladder extensions moved at the predicted virtual frequency of 2 Hz when applying the frame rate of 198.02 fps (Figs 4Ax-y, Bx-y, 5D-G, Supplementary Fig. S1Ax-y, Bx-y, S2Xl, Xr, Yl, Yr). Evaluation of displacements in micrometers obtained from the yt-reslicing and by using the outcomes of the template matching procedure yielded similar results (see below).

### Motion of isolated otoliths in agarose

As the two species show distinct differences in the shape of the saccular otoliths (fusiform in *S*. *tinanti vs*. rhomboid or pentagonal in *E*. *maculatus*; see also^31^), we first provide a brief description of the main otolith features. The saccular otoliths in both species show a heterosulcoid and ostial sulcus acusticus which is divided in an anterior portion (ostium) with an opening and a closed posterior portion (cauda). In *E*. *maculatus*, the rostrum (anterior portion) is longer and more distinctly pointed than in *S*. *tinanti*. The cauda is more strongly curved and more clearly delineated in *E*. *maculatus* than in *S*. *tinanti*. The crista superior forming the dorsal limit of the sulcus shows a crest-like structure almost along the entire length of sulcus in *E*. *maculatus*. In *S*. *tinanti*, the crista superior is less developed.

The estimated densities of the isolated saccular otoliths inferred from weight measurements and volumetry (Table 1) were close to the expected value for aragonitic otoliths of 2.93 g cm^−3 ^^3^, indicating that the tested otoliths were normally developed.

Sound impinging from above provoked otolith motion along the vertical (y-) axis (Fig. 4A0–2,B1–2,Ay,By; Supplementary Fig. S1Ay, By) but also led, to a lesser degree, to motion along the horizontal (x-) axis (Fig. 4Ax, Bx; Supplementary Fig. S1Ax, Bx). When sound impinged on the dorsal margin of the otolith, the saccular otoliths moved with a maximum displacement in y-direction of 20 µm to 32 µm in *E*. *maculatus* (anterior most otolith tip) and that of *S*. *tinanti* with 20 µm to 26 µm (anterior-most otolith tip and posterior margin). Displacement along the x-axis was less pronounced, with up to 6.5 µm in *E*. *maculatus* and 13 µm in *S*. *tinanti*. Along the x-axis, some parts of the otoliths in both species revealed a phase shift of ca. 90° relative to other parts within the same otolith. In the saccular otolith of *E*. *maculatus*, the crista superior moved differently to the rostrum and the posterior lobe (Fig. 4Ax). In the saccular otolith of *S*. *tinanti*, the phase shift occurred between the posterior margin relative to the rostrum and the crista superior (Supplementary Fig. S1Ax).

No such phase shift of otolith regions within one otolith was observed when sound impinged on the medial (sulcal) face of the saccular otolith. Otoliths moved in both x- and y-directions, with the otolith of *E*. *maculatus* displaying also a clear movement along the x-axis (Fig. 4Bx; Supplementary Movies S3–4). In this orientation, maximum otolith displacement in the two samples was 6.5 µm (rarely 13 µm) in both x- and y-directions.

For the *E*. *maculatus* otolith with sound impinging on the medial face, both analyzed bubbles revealed a clear phase shift compared to the otolith along the y-axis (Fig. 4By). This indicates a differential motion between the otolith and the surrounding medium (agarose). In contrast, the larger bubbles in the agarose block of the *S*. *tinanti* otolith moved in phase with the otolith in y-direction (S1By).

### Motion of otoliths *in-situ*

When sound impinged dorsally on the fish’s head, differential motion was observed for utricular and saccular otoliths. Utricular otoliths moved clearly along the x-axis and showed a slight swinging-like motion, whereas both saccular otoliths moved mainly in vertical (y-) direction (Supplementary Movie S5a).

For sound impinging on the right lateral body side of *E*. *maculatus*, a clear phase shift was detected for different structures of both body sides such as left and right otoliths (utricular, saccular, and lagenar otoliths), bones (parasphenoid, lower pharyngeal jaws, gill rakers), and both anterior swim bladder extensions in x-direction (Fig. 5D,F; Supplementary Movies S5a-b, Fig. S5c, S2Xl, Xr). In y-direction, in contrast, all measured structures of the left body side moved in phase (Fig. 5G; Supplementary Fig. S2Yl). On the right body side, a slight phase shift was observed in y-direction between the anterior swim bladder extension and the lagenar otolith on the one hand and the otoliths of the utricule and saccule on the other hand (Fig. 5E; Supplementary Fig. S2Yr).

Maximum displacements of otoliths as well as of bones and anterior swim bladder extensions were smaller (ca. 6.5 µm in x-direction, ca. 13 µm in y-direction) than those observed for isolated otoliths embedded in agarose.

## Discussion

The aim of our study was to test whether otolith motion can be visualized and evaluated *in-situ*. To our knowledge, we provide the first set-up and experimental data on the *in-situ* motion of otoliths in whole fish using a non-invasive synchrotron radiation imaging technique. According to our initial prediction, isolated otoliths in the simple agarose model and otoliths *in-situ* moved with a virtual frequency of 2 Hz. This strongly suggests that the settings used in our study depicted real motion due to the applied pure tone stimulus of 200 Hz.

### Otolith motion

The otoliths embedded in agarose apparently move as a “rigid” body, which is in accordance to the fact that otoliths are composed of more than 90% of rather inelastic calcium carbonate and only minor fractions of organic material (e.g.^37,38^). The observed 90° phase shifts within isolated otoliths of both studied species when sound impinged on the dorsal otolith margin are likely due to simultaneous motion of the otoliths along the third (here: medio-lateral) axis which has not been captured in our 2D recordings. Some parts such as the crista superior in the otolith of *E*. *maculatus* are located on another level along the medio-lateral axis than the rostrum and the posterior lobe. Accordingly, motion of the otolith along this axis may lead to just a virtual differential motion within one otolith. In addition, the saccular otoliths of the two species seem to show slight (mass- and/or shape-dependent) differences in their motion patterns. These assumptions could be tested by performing tomography that would capture otolith motion in its 3D aspect investigating larger sample sizes.

The amount of maximum displacement apparently depends on the position of the otolith in the sound field or relative to the position of the sound source. This interpretation is based on the fact that the isolated saccular otoliths of *E*. *maculatus* and *S*. *tinanti* embedded in agarose showed greater displacements when sound impinged on the dorsal otolith margin (“lateral” view) than on the medial otolith face (“dorsal” view). In “lateral” view, the otolith moves against less damping material (agarose), with the narrow dorsal margin of the otolith pointing into the direction of the sound source. In contrast, the resistance imposed by agarose to the greater area of the lateral otolith face may explain the smaller displacements in “dorsal” view. More damping by surrounding tissue and attached tissue (otolithic membrane) may also account for the smaller displacements of otoliths *in-situ*. Hence, measurements of the density of the endolymph and the otolithic membrane in the studied species are needed. Until now, data on the mechanical properties of the endolymph and the otolithic membrane are generally rare (e.g. Grant & Best^39^ indicating a density of 1.0 g/cm^3^ for both structures in the human “otolith organs”, i.e. utricle and saccule; see also^19^).

In the fish, the different otolith types, i.e. utricular, saccular, and lagenar otoliths, show different motion patterns as expected by their different *in-situ* orientation and amount of attachment to the respective underlying sensory epithelium. The utricular sensory epithelium is bowl-shaped and the respective otolith overlies it mainly along the horizontal plane (e.g.^9^). The utricular otolith thus has the most degrees of freedom for motion along the horizontal axes. This would be in line with the distinct motion of the utricular otolith along the x-axis in our experiment when sound impinged on the fish oriented with the dorsal side up. The sensory epithelia of the saccule and lagena are oriented along the dorso-ventral axis (e.g.^9^); in consequence, motion of the saccular and lagenar otoliths along the medio-lateral axis (i.e. y-axis in the recordings with sound impinging on the right body side) may be very limited; the dominant axis of motion in these otoliths is apparently along the fish’s dorso-ventral axis (i.e. y-axis in the recordings with sound impinging on the fish oriented with the dorsal side up) of the fish. These interpretations should be tested in further experiments using larger sample sizes, and otolith motion *in-situ* should also be studied in frontal view.

### Methodological aspects

In previous mathematical modelling, otolith displacement was calculated to be 0.6 µm in the rigid-sphere modelling^23^, which assumed a sound pressure of 1.0 kPa and a 200 Hz stimulus. In an elastic model that incorporated information about the stiffness of the ciliary bundles of sensory hair cells and the otolithic membrane^19^, otolith displacement was estimated to be 0.1 µm for an otolith of 1 mg mass at a frequency of 200 Hz and a sound pressure of 1.864 kPa. An experimental laser vibrometry study on perch^22^ found maximum otolith displacement at the otolith margins at 220 Hz with values up to 0.22 µm (in that study evaluated as a velocity of 150 µms^−1^); that experiment used a displacement of 0.91 µm produced by the shaker table set-up. Our study, using a sound pressure of 70.8 Pa with a maximum water displacement of 4 µm, yielded otolith displacements ranging from 6.5 µm to 32 µm. These values are distinctly larger than those obtained by modelling or in the former experimental study^22^. We used high SPLs and had to place the test subjects rather close to sound source due to the small tank size. Accordingly, the conditions in our set-up were far from those experienced by a fish in its natural habitat. This, however, was indispensable because we sought a methodological proof-of-principle and tried to provoke large displacements to ensure that available spatial and temporal resolutions would be sufficient to capture otolith motion, which we expected to be weak^22,23^.

Counter-directional methodological requirements of underwater acoustics and X-ray based imaging could be met only by a compromise. Assumptions based on ideal acoustic conditions (e.g. an infinite water body and a test object not too close to the sound source) would imply a large test tank. Conversely, the constraints imposed by the imaging, such as using less X-ray absorbent material demanded the smallest possible water body and a thin-walled test tank. Hence, the deviation between the calculated water displacement of 0.04 µm in our test tank – based on the assumption of ideal acoustic conditions and the experimentally evaluated value of maximally 4 µm – indicated more complex tank acoustics. To improve tank acoustics in future studies and to avoid potential enhancement effects that may account for the discrepancy between water displacement and the observed otolith displacements (4 µm versus up to 32 µm), two sound projectors could be used at both ends of a tube-shaped test tank. Two projectors would achieve a more homogeneous sound field (see^17^) along with more physiological sound pressure levels of less than 130 dB re 1 µPa (cf.^22^). Nevertheless, hard X-ray phase contrast imaging is a successful approach to visualize otolith motion in the simple agarose model as well as in whole fishes: the observed otolith motion almost perfectly matched the predicted virtual frequency of 2 Hz, and the differential displacements between structures (otoliths, bones, swim bladder) occurred in the intact tissue context when visualizing otolith motion *in-situ*.

Future experiments should also use different stimulus types referring to sounds produced by cichlids^40,41^, frequencies used for acoustic communication^42^, or related to the upper limits of hearing (ca. 0.7 kHz in *S*. *tinanti* and ca. 3 kHz in *E*. *maculatus*^30^). The stroboscopic imaging method used in our study could be scaled up to higher frequencies than 0.2 kHz as long as the studied system is periodic. Using sound stimuli of 0.7 kHz or 3 kHz would require integration times of 0.2857 ms or 0.0667 ms per radiogram, respectively. These values are 3.5 times and 15 times shorter than the integration time used in our study (1 ms) which would result in a worse signal-to-noise ratio per radiogram. This, however, might be improved by repeating the image acquisition at a given phase of motion and subsequently taking an average. By accumulating an equivalent total exposure time of 1 ms, the same image quality might be achieved as presented in this study.

## Conclusions

The set-up developed in our study including the simple model of an isolated otolith embedded in agarose, opens up a new direction of experimental investigation on the functional role of otoliths in the fish ear. Our approach provides the starting point for future experimental studies to yield fundamental data on factors influencing otolith motion. Synchrotron imaging techniques have constantly shifted limits towards higher spatial and temporal resolutions. Therefore, future experiments will also be able to elucidate otolith motion in 3D using phase contrast tomography.

## Electronic supplementary material

Supplementary information

Supplementary video S3

Supplementary video S4

Supplementary video S5a

Supplementary video S5b
