# Supplementary material for: In-situ visualization of sound-induced otolith motion using hard X-ray phase contrast imaging
Source: Sci Rep. 2018 Feb 15;8:3121. doi: 10.1038/s41598-018-21367-0 (PMC5814409; doi:10.1038/s41598-018-21367-0)
Supplement: Supplementary file 1 — Supplementary information [file 41598_2018_21367_MOESM1_ESM.doc]

In-situ visualization of sound-induced otolith motion using hard X-ray phase contrast imaging

Tanja Schulz-Mirbach^1^, Margie Olbinado^2^, Alexander Rack^2^, Alberto Mittone^3^, Alberto Bravin^3^, Roland R. Melzer^4^, Friedrich Ladich^5*^, Martin Heß^1^

^1^Ludwig-Maximilians-University Munich, Department Biology II, Zoology, Großhaderner Straße 2, 82152 Planegg-Martinsried, Germany

Phone +49 89 2180 6575 (TSM), +49 89 2180 74130 (MH)

[schulz-mirbach@biologie.uni-muenchen.de](mailto:schulz-mirbach@biologie.uni-muenchen.de), [hess@zi.biologie.uni-muenchen.de](mailto:hess@zi.biologie.uni-muenchen.de)

^2^European Synchrotron Radiation Facility (ESRF, ID19), 71 Avenue des Martyrs, 38000 Grenoble, France

Phone +33 476 88 1781 (AR)

[margie.olbinado@esrf.fr](mailto:margie.olbinado@esrf.fr), [alexander.rack@esrf.fr](mailto:alexander.rack@esrf.fr)

^3^European Synchrotron Radiation Facility (ESRF, ID17), 71 Avenue des Martyrs, 38000 Grenoble, France

Phone +33 476 88 2843 (AB)

[alberto.mittone@esrf.fr](mailto:alberto.mittone@esrf.fr), [bravin@esrf.fr](mailto:bravin@esrf.fr)

^4^Bavarian State Collection of Zoology (ZSM), Münchhausenstraße 21, 81247 Munich, Germany

Phone +49 89 8107 141

[melzer@zsm.mwn.de](mailto:melzer@zsm.mwn.de)

^5^University of Vienna, Department of Behavioural Biology, Althanstraße 14, 1090 Vienna, Austria

Phone +43 1 4277 54227

[friedrich.ladich@univie.ac.at](mailto:friedrich.ladich@univie.ac.at)

* corresponding author

**Legends to Supplementary Material**

**Fig.** **S1.** Motion of the saccular otolith of *S. tinanti* illustrated with residual plots of “displacement” versus image number (Ax-y, Bx-y) obtained by the template matching procedure in ImageJ v 1.51n. “A” indicates sound impinging on the otolith’s antero-dorsal margin, “B” on the medial face. All otolith regions move in phase along the y-axis in both views (Ay, By). For sound impinging on the medial face, no clear motion is visible along the x-axis. Out-of-phase motion occurs along the x-axis when sound impinged on the antero-dorsal otolith margin (Ax). The two bubbles and the otolith move in phase along the y-axis (By). Scale bar, 500 µm.

**Fig.** **S2.** Motion of otoliths, bones, and anterior swim bladder extensions in-situ in *E. maculatus* with sound impinging on the fish’s right body side. For selected regions used for the template matching procedure see Figure 5. Motion is illustrated with residual plots of “displacement” versus image number obtained by the template matching procedure in ImageJ v 1.51n. Plots in (Xr, Yr) show moving structures of the right body side, those in (Xl, Yl) moving structures of the left body side. For both body sides, otoliths, bones, and anterior swim bladder extensions move out-of-phase along the x-axis (Xl, Xr). Along the y-axis, all structures move in phase on the left body side (Yl), but slightly out-of-phase on the right body side (lagenar otolith and swim bladder vs. bones, utricular otolith, and saccular otolith; Yr).

**Movie.** **S3.** Moving saccular otolith (*E. maculatus*) embedded in 1% agarose shown in lateral view. (.mp4, frame rate: 198 fps)

**Movie.** **S4.** Moving saccular otolith (*S. tinanti*) embedded in 1% agarose shown in lateral view. (.mp4, frame rate: 198 fps)

**Movie. S5a.** Moving otoliths of *E. maculatus* in-situ shown in lateral view. In this view, both utricular and saccular otoliths, and the anterior swim bladder extensions are clearly visible. (.mp4, frame rate: 198 fps)

**Movie. S5b.** Moving right utricular otolith in dorsal view (same specimen as in S5a). (.avi, frame rate: 198 fps)

**Fig. S5c.** 3D reconstruction of (A) all otoliths of the same specimen as shown in S5a seen in lateral view and (B) of a utricular otolith of a similar sized individual as shown in S5a seen in dorsal (B1) and lateral (B2) views. Scale bars (A), 500 µm, (B), 100 µm.


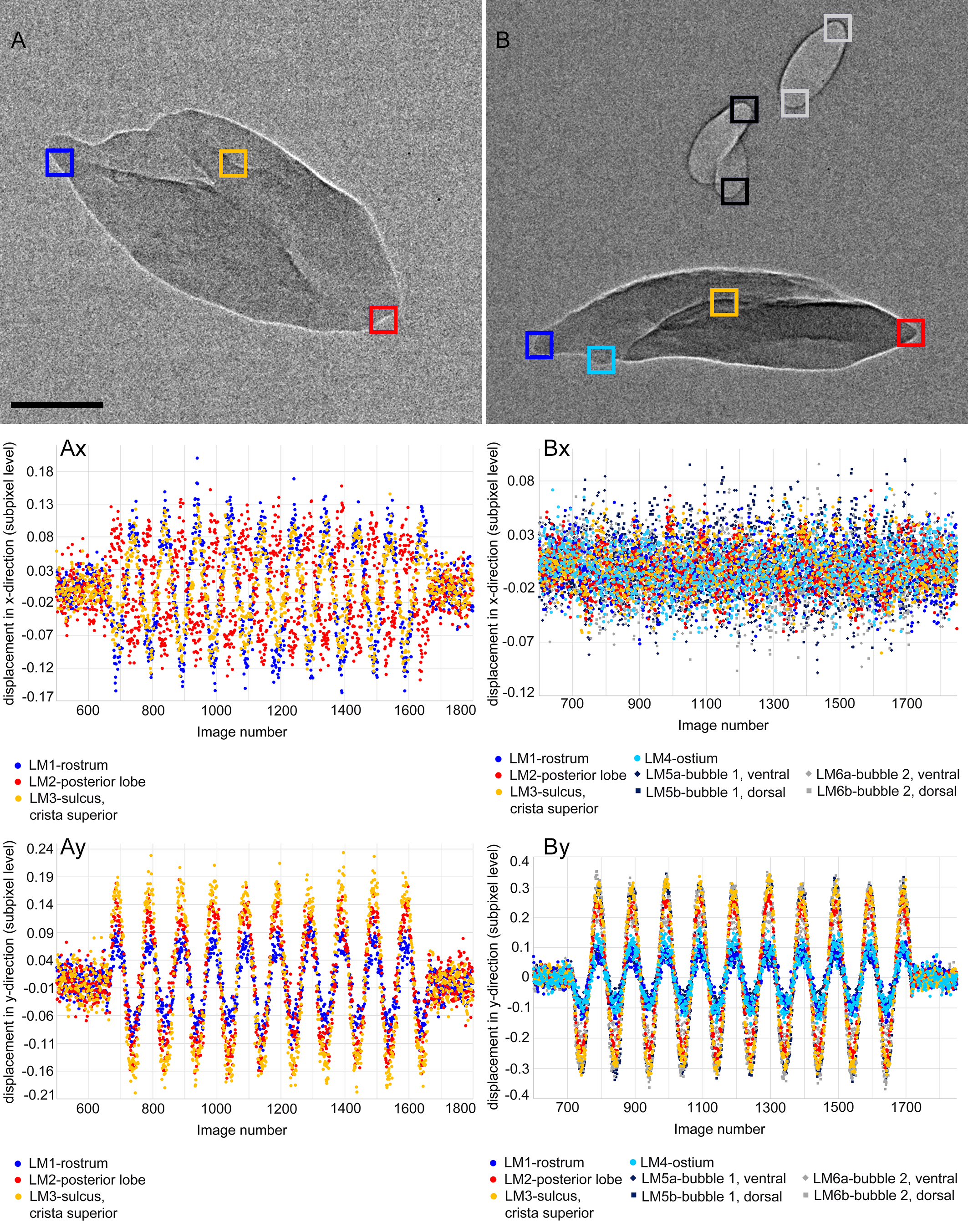


**Fig. S1**


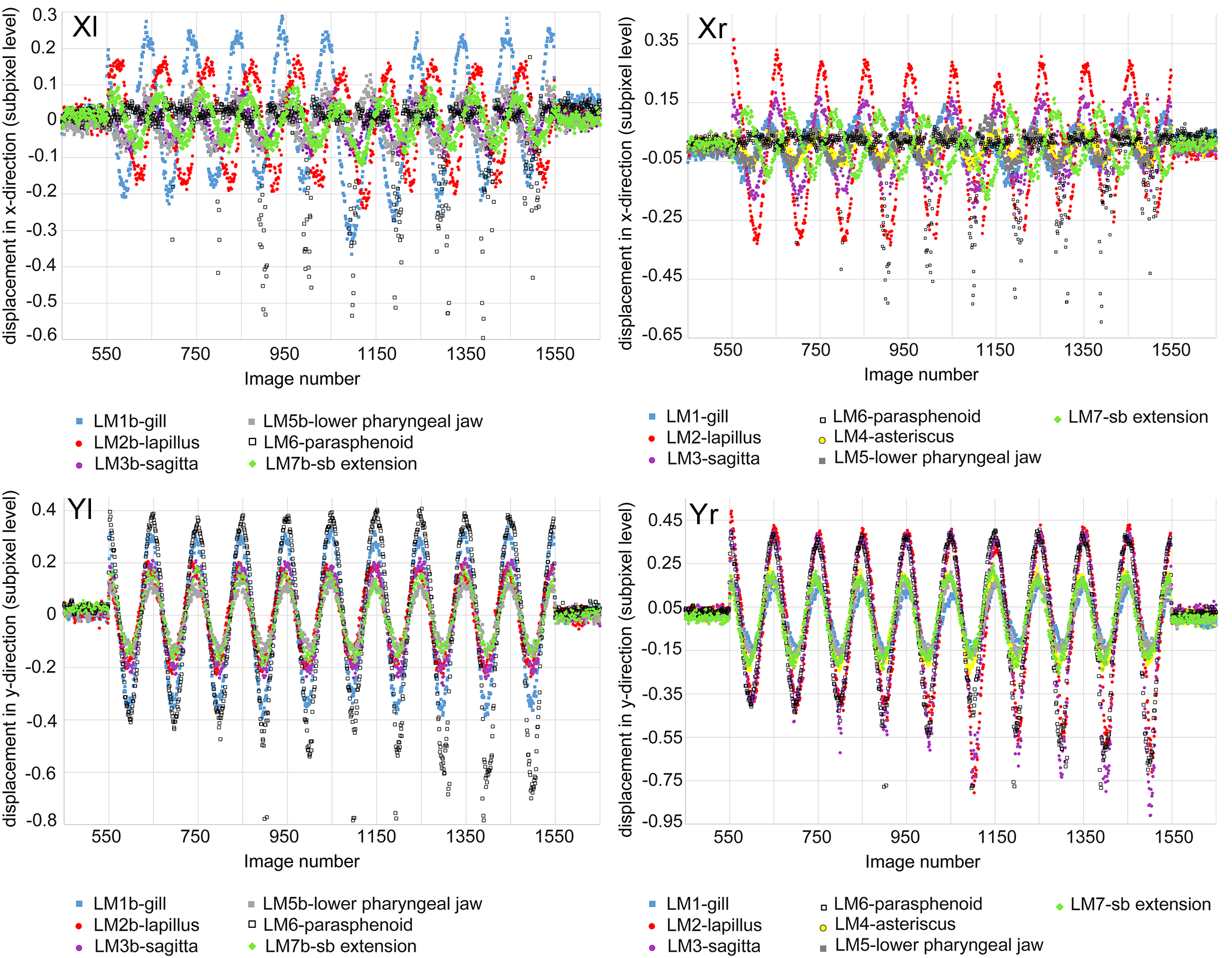


**Fig. S2**


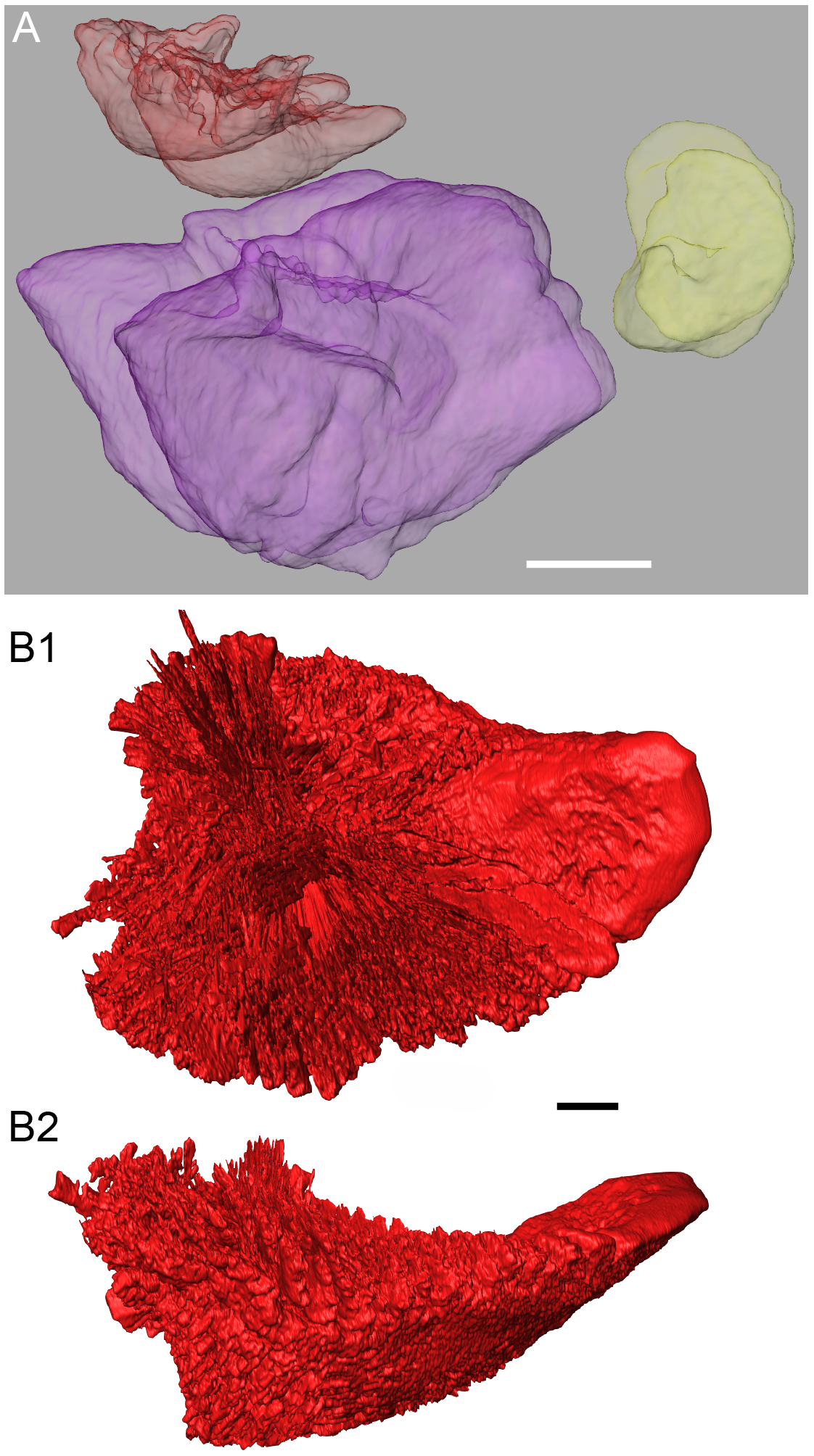


**Fig. S5c**
